# Sex differences in cardiac rehabilitation barriers among non-enrollees in the context of lower gender equality: a cross-sectional study

**DOI:** 10.1186/s12872-023-03331-7

**Published:** 2023-06-29

**Authors:** Mahdieh Ghanbari Firoozabadi, Masoud Mirzaei, Sherry L Grace, Mohammadreza Vafaeinasab, Maryam Dehghani-Tafti, Abbas Sadeghi, Zohre Asadi, Mohammad Hasan Basirinezhad

**Affiliations:** 1 Yazd Cardiovascular Research Center, Non-communicable Diseases Research Institute, Shahid Sadoughi University of Medical Sciences, Yazd, Iran; 2 Faculty of Health, York University, Toronto, ON Canada; 3 KITE- Toronto Rehabilitation Institute & Peter Munk Cardiac Centre, University Health Network, University of Toronto, Toronto, ON Canada; 4 Department of Nursing, Faculty of Nursing and Midwifery, Tehran Medical Sciences, Islamic Azad University, Tehran, Iran; 5 Department of Biostatistics and Epidemiology, School of Public Health, Shahid Sadoughi University of Medical Science, Yazd, Iran

**Keywords:** Ischemic heart disease, Cardiac rehabilitation, Access to care, Sex differences

## Abstract

**Background:**

Despite the benefits of cardiac rehabilitation (CR), it remains under-utilized, particularly by women. This study compared CR barriers between non-enrolling men and women in Iran, which has among the lowest gender equality globally.

**Methods:**

In this cross-sectional study, CR barriers were assessed via phone interview in phase II non-attenders from March 2017 to February 2018 with the Persian version of the Cardiac Rehabilitation Barriers Scale (CRBS-P). T-tests were used to compare scores, with each of 18 barriers scored out of 5, between men and women.

**Results:**

357 (33.9%) of the sample of 1053 were women, and they were older, less educated and less often employed than men. Total mean CRBS scores were significantly greater in women (2.37 ± 0.37) than men (2.29 ± 0.35; effect size[ES] = 0.08, confidence interval[CI]: 0.03–0.13; p < 0.001). The top CR barriers among women were cost (3.35; ES = 0.40, CI:0.23–0.56; P < 0.001), transportation problems (3.24; ES = 0.41, CI:0.25–0.58; P < 0.001), distance (3.21; ES = 0.31, CI:0.15–0.48; P < 0.001), comorbidities (2.97; ES = 0.49, CI:0.34–0.64; P < 0.001), low energy (2.41; ES = 0.29, CI:0.18–0.41; P < 0.001), finding exercise as tiring or painful (2.22; ES = 0.11, CI:0.02–0.21; P = 0.018), and older age (2.27; ES = 0.18, CI:0.07–0.28; P = 0.001). Men rated “already exercise at home or in community” (2.69; ES = 0.23, CI:0.1–0.36; P = 0.001), time constraints (2.18; ES = 0.15, CI:0.07–0.23; P < 0.001) and work responsibilities (2.24; ES = 0.16, CI:0.07–0.25; P = 0.001) as greater CR barriers than women.

**Conclusion:**

Women had greater barriers to CR participation than men. CR programs should be modified to address women’s needs. Home-based CR tailored to women’s exercise needs and preferences should be considered.

**Supplementary Information:**

The online version contains supplementary material available at 10.1186/s12872-023-03331-7.

## Background

Cardiovascular diseases are the most common burdens of disease worldwide [1], including in Iran [2]. Cardiac rehabilitation (CR) is a guideline-recommended model [3] of secondary prevention to mitigate this risk [4]. It generally involves patients travelling to a hospital two times per week over several months [5] for a tailored program of structured exercise, risk factor management as well as lifestyle and psychosocial counseling [6].

Despite the proven benefits of CR, these services are grossly under-used, even when compared to other guideline recommendations [7]. Moreover, certain subpopulations, often those who need it most, are even less likely to access CR [8]. For instance, meta-analyses reveal women are significantly less likely to be referred and enroll [9, 10], despite arguably greater need for many clinical (e.g., more comorbidity, lower functional capacity) and psychosocial (e.g., depression, lower socioeconomic status) reasons [11, 12], and the demonstrated benefit in women [13, 14]. There has been one study since which showed greater CR use in women [15]; This study was undertaken in Sweden, which is the 5th most gender equal country of the world, and the authors postulate in the discussion that this may explain the finding [16].

Barriers to participation in CR are multi-level: related to the health system (e.g., lack of programs, cost) [17], physicians (e.g., lack of referral) [18], the CR centers (e.g., hours, location), and to patients themselves (e.g., lack of motivation) [19].Recent reviews identified 24 studies assessing women’s CR barriers, which found many of the same above issues, but some that are more preponderant or unique to women (e.g., exercise pain or fatigue, comorbidities) [20]. Many of these studies however did not include male samples as a comparison, were qualitative or did not use a validated measure of barriers, had small sample sizes of women, and all were undertaken in “western” countries where gender inequality is relatively lower and hence barriers may be fewer [21].

Iran is a country with the 5th worst gender equality globally according to the World Economic Forum’s Global Gender Gap Index (rank = 148) [21], and indeed women are less likely to be referred and to participate in CR there [22]. The purpose of this study was to quantitatively compare CR barriers to participation in CR between men and women who were referred to CR but did not enroll in this country.

## Materials and methods

### Design and setting

This cross-sectional study was conducted in Afshar CR center, Yazd, central Iran, which is a referral centre for the southern provinces of Iran. Secondary analysis of data from a previous study of inpatient CR participants who did not attend phase II CR was undertaken [23]. The Ethics Committee of Shahid Sadoughi University of Medical Sciences, Yazd, Iran approved the study (no.IR.SSU.MEDECINE.REC.1396.51). Informed consent was obtained from all study participants or their legally-acceptable representative where applicable. All the study procedures were conducted following the Declaration of Helsinki.

The CR team or inpatient nurse educates inpatients about the outpatient program and encourages them to attend. Written materials about CR are made available to cardiologists, but the proportion giving them to patients is unknown. At the time of the study, 60% of inpatient CR participants were referred to the outpatient program, and the participation rate was 6.9% (unfortunately since the study the inpatient CR program has closed) [24].

There is no waiting list for the phase II program. Afshar CR center is in a public institution. CR is covered by workplace healthcare insurance where available [25], The cost is about 1,670,000 Rials ($12). For those who have insurance, 70% of the cost is covered. The program is 36 sessions as is delivered in the United States [24]. It has a flexible schedule for employed patients, who are provided a certificate of attendance for their workplace. The program is lead by a Medical Director. The CR program has single-sex classes [26]. There was no home-based CR model available at the time of the study. Although it is available, it is difficult to reach and access the centre via public transportation, and there is no parking by the centre.

### Participants

The target population included post-myocardial infarction, post-coronary revascularization, acute coronary syndrome (ACS), and heart failure (HF) inpatients that were referred by their cardiologists to CR, and had not presented to the outpatient CR center at their appointed time between March 2017 and February 2018. The inclusion criteria were: cardiac patients without any other condition that would preclude them from participating in CR. The exclusion criteria were: living more than 50 km (31.1 miles) from the CR centre, particularly given there are no other proximate CR centres which they could attend.

### Procedure and Measures

CR nurses administered a questionnaire comprising two parts by telephone interview. Sociodemographic variables assessed included age, work status, sex, educational level, marital status, and number of children. To evaluate CR barriers between men and women, the Persian version of the Cardiac Rehabilitation Barriers Scale (CRBS-P) was used [23].

The CRBS evaluates patients’ multi-level barriers to CR enrolment and participation. The original English version consists of 21 items [21]. Scale items are rated on a 5-point Likert-type scale that ranges from 1 (strongly disagree) to 5 (strongly agree), with higher mean scores indicating greater barriers. This scale was translated and validated by Ghanbari-Firoozabadi et al. to Persian, and comprises 18 items [23]; the items about not knowing about CR, not needing CR and severe weather were removed for cultural adaptation. The scale consists of four subscales: perceived need/healthcare factors, logistical factors, comorbidities/functional status, and work/time conflicts, which are consistent between translations. The CRBS-P is highly reliable, with demonstrated content and construct validity [23].

### Statistical analyses

All data analyses were performed using SPSS Ver. 19.0. Descriptive statistics, such as mean and standard deviation (SD), as well as frequency and valid percentage were used to typify participants’ characteristics. Chi-squared, Fisher’s Exact, and independent samples *t*-tests were used to compare characteristics between men and women.

The Kolmogorov-Smirnov test was used to confirm normal distribution of CRBS responses. Thus, to test for sex differences in CRBS-P items, *t*-tests were used. A P-value of < 0.05 was considered statistically significant, but effect sizes with corresponding confidence intervals were also computed.

## Results

Participant characteristics are shown in Table 1. Among the 1053 participants, 357 (33.9%) were female, and the female participants were significantly older, less educated, less likely to be employed and had more children than male participants. Most female participants had ACS and men coronary revascularization, and this also significantly differed.

**Table 1:** Participant Characteristics

n (%)	Male (n = 696)		Female (n = 357)	*P*
**Age** (mean ± SD)	61.35 ± 12.57		65.24 ± 11.83	< 0.001
**Marital status**				0.829
Married		691 (99.3)	354 (99.2)	
Single/ divorced / widowed		5 (0.7)	3 (0.8)	
**Educational level**				< 0.001
Illiterate		279 (40.1)	245 (68.6)	
Primary school		227 (32.6)	87 (24.4)	
High school		103 (14.8)	16 (4.5)	
Associate’s / bachelor’s degree		76 (10.9)	8 (2.2)	
Master’s degree or above		11 (1.6)	1 (0.3)	
**Work status**				< 0.001
No paid employment		310 (44.5)	347 (97.2)	
Employed		386 (55.5)	10 (2.8)	
**Number of children (**mean ± SD)		4.37 ± 2.11	5.26 ± 2.24	< 0.001
**Cardiac diagnoses/ procedures**				< 0.001
ACS		157 (22.6)	145 (40.6)	
Revascularization		280 (40.2)	118 (33.1)	
MI		201 (28.9)	65 (18.2)	
HF	58 (8.3)		29 (8.1)	

**ACS**, acute coronary syndrome; **MI**, post-myocardial infarction; **HF**, heart failure

### CR barriers by sex

Mean CRBS-P scores are shown in Table 2, and as displayed, women had significantly higher barriers than men overall. With regard to the 4 subscales, women scored significantly higher on 2 of the subscales, namely logistical factors and comorbidities / functional status, and men scored higher on one, namely work/time conflicts.

**Table 2:** CR Barriers by Sex

CRBS-P Subscales/Items	Male (n = 696)	Female (n = 357)	Total (n = 1053)	P	Effect Size	95% Confidence Interval of the Difference	
						Lower	Upper
**Logistical factors**	2.72 ± 0.90	3.01 ± 0.97	2.82 ± 0.93	< 0.001	-0.23	-0.40	-0.16
1-Distance	2.89 ± 1.26	3.21 ± 1.35	3.00 ± 1.30	< 0.001	-0.31	-0.48	-0.15
2-Cost	2.95 ± 1.25	3.35 ± 1.30	3.09 ± 1.28	< 0.001	-0.40	-0.56	-0.23
3-Transportation problems	2.82 ± 1.24	3.24 ± 1.32	2.96 ± 1.28	< 0.001	-0.41	-0.58	-0.25
4-Family responsibilities	2.26 ± 0.89	2.26 ± 0.91	2.26 ± 0.90	0.930	0.01	-0.11	0.12
**Work/time conflicts**	2.14 ± 0.58	2.02 ± 0.42	2.10 ± 0.53	< 0.001	0.11	0.05	0.17
7-Travel	2.01 ± 0.58	1.98 ± 0.43	2.00 ± 0.53	0.246	0.03	-0.02	-0.09
8-Time constraints	2.18 ± 0.81	2.02 ± 0.53	2.12 ± 0.73	< 0.001	0.15	-0.07	0.23
9-Work responsibilities	2.24 ± 0.86	2.07 ± 0.63	2.18 ± 0.80	0.001	0.16	0.07	0.25
**Comorbidities/functional status**	2.19 ± 0.63	2.46 ± 0.70	2.28 ± 0.67	< 0.001	-0.27	-0.36	-0.18
6-I find exercise tiring or painful	2.10 ± 0.69	2.22 ± 0.79	2.14 ± 0.73	0.018	-0.11	-0.21	-0.02
10-I don’t have energy	2.11 ± 0.73	2.41 ± 0.96	2.22 ± 0.83	< 0.001	-0.29	-0.41	-0.18
11-Other health problems prevent me from going	2.47 ± 1.06	2.97 ± 1.22	2.64 ± 1.14	< 0.001	-0.49	-0.64	-0.34
12-I am too old	2.09 ± 0.70	2.27 ± 0.86	2.15 ± 0.76	0.001	-0.18	-0.28	-0.07
**Perceived need/healthcare factors**	2.15 ± 0.40	2.11 ± 0.38	2.14 ± 0.39	0.058	0.04	-0.00	0.09
5-I already exercise at home, or in my community	2.69 ± 1.10	2.46 ± 0.99	2.61 ± 1.07	0.001	0.23	0.10	0.36
13-My cardiologist or thoracic surgeon didn’t feel it was necessary	2.15 ± 0.70	2.15 ± 0.66	2.15 ± 0.69	0.956	0.01	-0.08	0.09
14-Many people with heart problems don’t go, and they are fine	2.06 ± 0.47	2.02 ± 0.38	2.05 ± 0.44	0.151	0.03	-0.01	0.09
15-I can manage my heart problems on my own	2.10 ± 0.55	2.05 ± 0.46	2.08 ± 0.52	0.084	0.05	-0.01	0.11
16-I think I was referred, but the rehab program didn’t contact me	2.03 ± 0.43	2.04 ± 0.41	2.04 ± 0.42	0.944	-0.01	-0.05	0.05
17-It took too long to start the outpatient program after referral	2.03 ± 0.40	2.03 ± 0.39	2.03 ± 0.40	0.937	-0.01	-0.05	0.04
18-I prefer to take care of my health alone, not in a group	2.04 ± 0.47	2.03 ± 0.43	2.04 ± 0.44	0.640	0.01	-0.04	0.07
**Total**	2.29 ± 0.35	2.37 ± 0.37	2.32 ± 0.36	< 0.001	-0.08	-0.13	-0.03

Mean ± standard deviation shown. CRBS-P, cardiac rehabilitation barriers scale, Persian

As also shown in Table 2, women’s greatest barriers were cost, transportation, distance and comorbidities; their lowest barriers were travel, time constraints, perceiving it is not needed, preferring to self-manage and wait time. Men’s greatest barriers were cost, distance, transportation, and already exercising; their lowest barriers were travel, wait time, lack of follow-up from the CR program and preferring to self-manage.

Table 2 also displays sex differences by CRBS item. Distance, cost, transportation problems, finding exercise as tiring or painful, low energy, health problems, and older age items were rated significantly higher by women. Among men, already exercise at home or in community, time constraints, and work responsibilities were significantly greater barriers to CR participation than they were among women. CRBS scores are shown by item by cardiac indication and sex in the online Additional File Table 1.

## Discussion

There are few studies of CR barriers outside of western high-income settings [27], and none to our knowledge quantitively assessing sex differences in these barriers using a validated scale in a less gender-equal society. In this first such study in a relatively large sample of Iranian non-enrollees, key barriers were quite consistent with what is reported in the broader literature (i.e., cost, distance, transportation), and sex differences were numerous. Women reported significantly greater barriers to CR participation than men.

The results can be placed within the context of the literature. To our knowledge, the CRBS and CR Enrolment Obstacles (CREO) scales are the only validated quantitative measures of CR barriers [28]. Following a rapid review of the literature, we could only identify one other study using one of these measures to assess sex differences in CR barriers, which was undertaken in Canada. There the context is different: Canada is ranked 19th on the Global Gender Gap Index [29], and CR is fully reimbursed, including widely-available home-based services [30]. In that study, there was no significant difference in total CR barriers, but similar to this study, there were significant sex differences for individual barrier items, with some greater in women and some in men. For instance, in both studies [29], men rated already exercising at home or in the community greater than women. While men are generally more physically active than women [31], we must educate all patients that CR is more than just exercise [6], and that the benefits are great.

Women’s top barriers can be compared and contrasted between the studies. In that other Canadian sex difference barrier study [29], women’s top barriers were not logistical as they were herein, but instead not knowing about CR (this item was not included in the translation; [23]) or being encouraged by their physician (this item was somewhat differently worded in the translation) were top; however, finding exercise tiring or painful and already exercising at home were also great barriers in both studies. This suggests that, in a lower-resource setting, logistical barriers are paramount as is poor health for women. The majority of women were unemployed (hence why work responsibilities were a greater barrier in men than women) and therefore had no independent income, thus the costs of CR sessions, and indirect costs such as transportation to the CR center were formidable barriers. Women lacked financial independence to pay for transportation, or to have a personal vehicle. And in the Iranian province where the study was undertaken there is only this one CR center, so distance was an issue.

With regard to sex differences, similarly between these only two studies of sex differences in CR barriers [29], women rated transportation higher, but also perceiving exercise as tiring or painful and comorbidities as higher barriers than men. Many studies have documented women’s lower functional capacity upon CR entry, as well as their musculoskeletal [32] and other comorbidities which may hinder their full participation in CR [33, 34], but in most cases are actually also ameliorated by it.

Finally, with regard to sex differences contrary to this study, in the Canadian study and others[35], women also rated family obligations higher than men. One of the possible reasons for the lack of difference in this barrier between men and women in this study may be that many men were retired and thus spend more time on household responsibilities and family care [36]. Another reason for this difference may be that women’s children had grown, and as they were not working, they perceived fewer conflicts related to this life role.

## Implications

A review of women’s CR barriers and associated solutions sheds light on potential strategies to overcome these barriers [37]. Many of the solutions focus on the referral process itself and automating it including an encouraging discussion with patients, which is in place at this center but could be bolstered. A recent update of the Cochrane review on interventions to increase CR utilization also suggests home-based models could work [5]. Indeed, a home-based model has been initiated since the COVID-19 pandemic, via phone with free follow-up monitoring. Offering “women-focused” CR may also increase utilization [38], and that is already offered at the center under study (i.e., it is women-only), although more tailoring to better meet women’s needs may be warranted [39]. For instance, the recent International Council of Cardiovascular Prevention and Rehabilitation clinical practice guideline [39] on this matter recommends careful attention to comorbidities as well as feelings of pain and fatigue when developing, initiating and progressing women’s exercise prescriptions.

Finally, in an effort to move the field from assessment of barriers to mitigation of them, the CRBS is now available online in several languages for patients to complete, and strategies to address their top-rated barriers are provided (https://globalcardiacrehab.com/For-Patients). We are now testing the value of the responses and seeking to optimize them to be applicable across a broad range of settings, and then will test impact on utilization. Ultimately however, we must tackle all levels at play: not just the patient level, but increasing capacity (i.e., health system level), ensuring referral (i.e., physician level), and optimizing accessibility of programs (i.e., center level).

### Study limitations

Caution is warranted in interpreting these results. First, the present study was undertaken in a single center, such that generalizability to other centers, including those in other lower-resource, gender-unequal, Muslim settings is not known. Future research in other such settings is warranted. Second, it is unknown whether response or selection bias was at play, as reasons for declining participation were not collected from non-consenting patients. Third, there could be inflated error due to multiple comparisons. Finally, due to the nature of the study design, causal conclusions cannot be drawn.

## Conclusions

In this gender-unequal lower-resource context, women had significantly greater CR barriers than men, and the nature of their barriers differ from those of women in higher-resource settings. Their greatest barriers among women were logistical factors (distance, cost, and transportation problems) and comorbidities/functional status (such as finding exercise as tiring or painful, low energy level, health problems, and older age). Alternative models such as home-based CR should be considered for patients who have such logistical barriers. Overcoming cost and transportation barriers requires women to have more economic resources. Finally, patients must be informed their CR exercise prescriptions will be tailored based on their functional status, comorbidities, and preferences.

## Electronic supplementary material

Below is the link to the electronic supplementary material.

Additional File 1 Table 1: Cardiac rehabilitation barriers (organized by subscale) by cardiac indication and sex

## Data Availability

The datasets used and/or analyzed during the current study are available from the corresponding author on reasonable request.

## Abbreviations

CR: Cardiac rehabilitation
CRBS: Cardiac Rehabilitation Barriers Scale
CRBS-P: Persian version of the Cardiac Rehabilitation Barriers Scale
ACS: Acute coronary syndrome
HF: Heart failure
SD: Standard deviation
