# Correction: Sex differences in cardiac rehabilitation barriers among non-enrollees in the context of lower gender equality: a cross-sectional study

**DOI:** 10.1186/s12872-023-03385-7

**Published:** 2023-07-17

**Authors:** Mahdieh Ghanbari Firoozabadi, Masoud Mirzaei, Sherry L Grace, Mohammadreza Vafaeinasab, Maryam Dehghani-Tafti, Abbas Sadeghi, Zohre Asadi, Mohammad Hasan Basirinezhad

**Affiliations:** 1 Yazd Cardiovascular Research Center, Non-communicable Diseases Research Institute, Shahid Sadoughi University of Medical Sciences, Yazd, Iran; 2 Faculty of Health, York University, Toronto, ON Canada; 3 KITE- Toronto Rehabilitation Institute & Peter Munk Cardiac Centre, University Health Network, University of Toronto, Toronto, ON Canada; 4 Department of Nursing, Faculty of Nursing and Midwifery, Tehran Medical Sciences, Islamic Azad University, Tehran, Iran; 5 Department of Biostatistics and Epidemiology, School of Public Health, Shahid Sadoughi University of Medical Sciences, Yazd, Iran

Following publication of the original article [1], in this article the affiliation details for Authors Maryam Dehghani-Tafti and Mohammad Hasan Basirinezhad were swapped and the affialtion should read as follow:

^1^Yazd Cardiovascular Research Center, Non-communicable Diseases Research Institute, Shahid Sadoughi University of Medical Sciences, Yazd,Iran

^2^Faculty of Health, York University, Toronto, ON, Canada

^3^KITE- Toronto Rehabilitation Institute & Peter Munk Cardiac Centre, University Health Network, University of Toronto, Toronto, ON, Canada

^4^Department of Nursing, Faculty of Nursing and Midwifery, Tehran Medical Sciences, Islamic Azad University, Tehran, Iran

^5^Department of Biostatistics and Epidemiology, School of Public Health, Shahid Sadoughi University of Medical Sciences, Yazd, Iran

The original article has been corrected.
