# Supplementary material for: Sex differences in cardiac rehabilitation barriers among non-enrollees in the context of lower gender equality: a cross-sectional study
Source: BMC Cardiovasc Disord. 2023 Jun 29;23:329. doi: 10.1186/s12872-023-03331-7 (PMC10311813; doi:10.1186/s12872-023-03331-7)
Supplement: Supplementary file 1 — Additional File 1 Table 1: Cardiac rehabilitation barriers (organized by subscale) by cardiac indication and sex [file 12872_2023_3331_MOESM1_ESM.doc]

**Supplemental Table:** Cardiac rehabilitation barriers (organized by subscale) by cardiac indication and sex

| **CRBS-P Subscales/**Items | **ACS** | | **Revascularization** | | **MI** | | **HF** | |
| --- | --- | --- | --- | --- | --- | --- | --- | --- |
|  | **Male**  (n = 157) | **Female**  (n =145) | **Male**  (n =280) | **Female**  (n =118) | **Male**  (n =201) | **Female**  (n =65) | **Male**  (n =58) | **Female**  (n =29) |
| **Logistical factors** | 2.68±0.95 | 3.02±0.95^**^ | 2.72±0.82 | 3.11±0.92^***^ | 2.70±0.93 | 2.76±1.09 | 2.96±0.98 | 3.10±0.96 |
| 1-Distance | 2.77±1.33 | 3.20±1.31^**^ | 2.94±1.21 | 3.31±1.35^*^ | 2.81±1.27 | 2.98±1.46 | 3.21±1.30 | 3.34±1.37 |
| 2-Cost | 2.89±1.30 | 3.34±1.27^**^ | 2.91±1.19 | 3.53±1.27^***^ | 2.99±1.29 | 2.97±1.39 | 3.21±1.30 | 3.52±1.27 |
| 3-Transportation problems | 2.84±1.32 | 3.25±1.31^**^ | 2.75±1.15 | 3.36±1.30^***^ | 2.75±1.26 | 2.89±1.39 | 3.31±1.28 | 3.45±1.29 |
| 4-Family responsibilities | 2.23±0.95 | 2.30±0.98 | 2.30±0.83 | 2.25±0.83 | 2.28±0.96 | 2.23±0.96 | 2.14±0.76 | 2.10±0.72 |
| **Work/time conflicts** | 2.06±0.64 | 2.02±0.45 | 2.20±0.50^**^ | 2.07±0.39 | 2.16±0.65^*^ | 1.98±0.45 | 1.97±0.45 | 1.93±0.25 |
| 7-Travel | 1.99±0.71 | 1.99±0.47 | 2.08±0.53 | 2.03±0.45 | 1.96±0.55 | 1.88±0.33 | 1.97±0.49 | 1.93±0.25 |
| 8-Time constraints | 2.06±0.79 | 2.01±0.51 | 2.24±0.77^*^ | 2.06±0.57 | 2.24±0.94^*^ | 2.02±0.59 | 1.93±0.41 | 1.93±0.25 |
| 9-Work responsibilities | 2.15±0.86 | 2.07±0.66 | 2.28±0.80^*^ | 2.12±0.64 | 2.30±1.00^*^ | 2.06±0.65 | 1.93±0.41 | 1.93±0.25 |
| **Comorbidities/functional status** | 2.17±0.70 | 2.51±0.75^***^ | 2.17±0.45 | 2.37±0.55^**^ | 2.09±0.62 | 2.41±0.70^**^ | 2.68±0.93 | 2.78±0.85 |
| 6-I find exercise tiring or painful | 2.10±0.79 | 2.31±0.86^*^ | 2.04±0.46 | 2.14±0.61 | 2.07±0.72 | 2.03±0.72 | 2.57±1.04 | 2.52±1.05 |
| 10-I don’t have energy | 2.11±0.83 | 2.49±1.02^***^ | 2.10±0.58 | 2.31±0.88^*^ | 2.00±0.64 | 2.31±0.91^*^ | 2.64±1.11 | 2.66±1.11 |
| 11-Other health problems prevent me from going | 2.43±1.11 | 2.86±1.16^***^ | 2.49±1.02 | 2.93±1.19^**^ | 2.32±1.01 | 3.06±1.37^***^ | 3.03±1.16 | 3.48±1.15 |
| 12-I am too old | 2.09±0.85 | 2.39±0.95^**^ | 2.07±0.54 | 2.09±0.57 | 2.00±0.61 | 2.25±1.00 | 2.48±1.10 | 2.48±0.98 |
| **Perceived need/healthcare factors** | 2.10±0.45 | 2.10±0.34 | 2.18±0.36 | 2.11±0.37 | 2.17±0.43 | 2.10±0.48 | 2.12±0.25 | 2.13±0.31 |
| 5-I already exercise at home, or in my community | 2.52±1.10 | 2.39±0.95 | 2.88±1.08^*^ | 2.62±1.02 | 2.62±1.10 | 2.49±1.10 | 2.53±1.03^*^ | 2.10±0.72 |
| 13-My cardiologist or thoracic surgeon didn’t feel it was necessary | 2.24±0.92 | 2.10±0.57 | 2.08±0.48 | 2.11±0.56 | 2.18±0.76 | 2.23±0.89 | 2.19±0.63 | 2.38±0.77 |
| 14-Many people with heart problems don’t go, and they are fine | 1.98±0.48 | 2.03±0.39 | 2.09±0.43 | 2.02±0.36 | 2.09±0.54 | 1.98±0.41 | 2.05±0.34 | 2.07±0.37 |
| 15-I can manage my heart problems on my own | 2.04±0.58 | 2.06±0.47 | 2.11±0.49 | 2.03±0.41 | 2.15±0.63^*^ | 1.98±0.48 | 2.07±0.41 | 2.17±0.53 |
| 16-I think I was referred, but the rehab program didn’t contact me | 1.97±0.44 | 2.05±0.39 | 2.05±0.34 | 2.01±0.33 | 2.07±0.53 | 2.03±0.58 | 2.02±0.35 | 2.10±0.40 |
| 17-It took too long to start the outpatient program after referral | 1.99±0.48 | 2.06±0.38 | 2.05±0.33 | 2.01±0.33 | 2.03±0.45 | 2.00±0.50 | 2.02±0.35 | 2.07±0.37 |
| 18-I prefer to take care of my health alone, not in a group | 1.97±0.50 | 2.05±0.39 | 2.06±0.40 | 2.01±0.35 | 2.08±0.55 | 2.00±0.50 | 2.02±0.35 | 2.07±0.37 |
| **Total** | 2.24±0.40 | 2.38±0.36^**^ | 2.30±0.29 | 2.38±0.33^*^ | 2.27±0.37 | 2.30±0.45 | 2.41±0.32 | 2.46±0.31 |

Mean ± standard deviation shown. CRBS-P, cardiac rehabilitation barriers scale, Persian.

**ACS,** acute coronary syndrome; **MI,** myocardial infarction; **HF,** heart failure

^*^ Significant difference between male and female participants based on t-tests; ^*^ p < 0.05; ^**^ p < 0.01; ^***^ p < 0.001
